# Enhancing team resilience through team knowledge dynamics: a mediated approach

**DOI:** 10.3389/fpsyg.2025.1615909

**Published:** 2025-09-04

**Authors:** Hong Yang, Sid Suntrayuth

**Affiliations:** International College, National Institute of Development Administration, Bangkok, Thailand

**Keywords:** team dynamic capability, knowledge creation, team resilience, wise leadership, communicative, interpretation, analytical

## Abstract

In rapidly changing and knowledge-driven environments, building resilient teams has become a strategic imperative for knowledge-based organizations, particularly higher education institutions. This paper conceptualizes team dynamic capabilities using the SECI model and examines the serial mediating roles of communicative, leadership, interpretation, and analytical. Using survey data from 617 faculty and staff members in Chinese higher education institutions. The study applies structural equation modeling (SEM) to test the proposed relationships. The results show that these four behavioral capacities serve as partial mediators between team dynamic capability and team resilience, highlighting the importance of knowledge-based team processes in shaping resilience. The findings provide practical implications for higher education institutions in building resilient teams under changing environments.

## 1 Introduction

Higher education institutions worldwide face diverse pressures originating from both inside and outside the university, from society, industry, and politics (Young et al., 2024). In recent years, universities worldwide have faced increasing pressure to adapt to rapidly changing environments, both externally and internally. In addition to external environmental pressures, functionalist expectations demand that universities broaden and extend their missions to meet the evolving needs of diverse stakeholders (Compagnucci and Spigarelli, 2020; Enders and De Boer, 2009). Within the Chinese higher education system, public universities are characterized by a pronounced hierarchical structure, including national, provincial, and local institutions. This stratification has been further intensified by the “Double World-Class Project,” a national policy that concentrates resources on a select group of elite universities and disciplines to enhance global competitiveness. Consequently, local universities that are excluded from this policy framework face mounting transformational pressures. These institutions are compelled to pursue differentiated development strategies and institutional innovations to sustain their relevance and viability.

Meanwhile, since 2019, China’s higher education system has transitioned from a stage of massification to universalization, shifting its reform focus toward high-quality development, particularly in terms of innovation potential. Innovation has been increasingly recognized as a value-added driver of higher education institutions’ social contributions (Huizingh, 2017), and as a means of continuously stimulating institutional vitality (Liu, 2021). These evolving demands have placed pressure on higher education systems to cultivate resilience and build adaptive capacities to respond to change. Resilience enables higher education institutions to maintain quality and operational continuity amid disruptions (Karlsson and Offord, 2023; Ramirez, 2020). Although universities have historically been viewed as among the most resilient organizations in society (Stolze and Sailer, 2022), there is now a global call for higher education to adopt new models and practices—developing management capabilities once reserved for businesses (Stolze and Sailer, 2022).

In the context of transformation, local higher education institutions often operate in environments disrupted by shifts in national budgets, new governance mandates, and even political interventions (Teece, 2018). especially when academic staff lack research and development skills, motivation, and awareness of knowledge creation. At the same time, the challenges faced by institutions are often characterized by a high degree of uncertainty, systemic interdependence, and complexity, which require a higher level of organizational coordination and knowledge integration mechanisms. Against this backdrop, teams have become increasingly important as basic organizational units within universities, serving to connect individuals, integrate knowledge, and drive task execution. Teams are not only carriers of knowledge and resource flows, but also key spaces for implementing management objectives and building organizational resilience. Therefore, a team’s ability to maintain coordination, flexibility, and adaptability under changing external environments has become a critical factor affecting the overall operational quality and transformation outcomes of higher education institutions. A prevalent feature of internal operations in Chinese higher education institutions is the dual role system, under which faculty members often assume multiple responsibilities across teaching, research, and administrative functions. This policy-driven structure significantly shapes the nature of academic teams, resulting in incomplete team compositions and blurred team boundaries. Within such a context, collaboration tends to be highly cross-departmental and interdisciplinary. If stakeholders can effectively mobilize interactive resources and establish stable mechanisms of cooperation, it may unlock greater collaborative potential and contribute to the generation of enhanced public value.

However, university teams currently face a major challenge in responding to complex tasks—namely, how to effectively improve problem-solving capabilities. Although team collaboration is believed to enhance problem-solving ability (De Boeck and Scalise, 2019), in practice, team members often encounter multiple challenges that may interrupt workflows and reduce performance, thereby weakening team resilience (Stoverink et al., 2020). These challenges include conflict management (Fisher et al., 2020), sustaining trust (Newman and Ford, 2021), overcoming communication barriers (Casoria et al., 2020), and maintaining accountability (Stewart et al., 2023). In other words, even when various collaborative f mechanisms are in place, teams may still fail due to internal obstacles (Tannenbaum et al., 2012).

Owing to the various potential barriers to team collaboration, team dynamics can help teams overcome these challenges and unlock their potential (Neves, 2024). In fact, in diverse practical contexts, the complementary skills of a team members are essential for exponentially accelerating an organization’s overall success (Neves, 2024). Especially in a knowledge economy environment, the value of team collaboration lies in its absorptive capacity for diverse perspectives and specialized knowledge (Thayer et al., 2018). According to the dynamic capability theory, the generative aspect of capabilities stems from team-level interactions among organizational members. When such interactions can identify and seize opportunities presented in the form of knowledge, they can lead to resource recombination and transformation. Within the dynamic capability framework, knowledge-based opportunities that are perceived and captured can result in the reconfiguration of existing resources (Nonaka et al., 2016). In this process, the knowledge creation model (Socialization–Externalization–Combination–Internalization, SECI) (Nonaka et al., 1996) provides a theoretical lens to understand how teams create knowledge through dynamic interaction, supporting the continuous evolution of knowledge and the reconstruction of capabilities. It offers important theoretical support for understanding how team members engage in knowledge creation. Existing research suggests that resilience is jointly determined by endogenous sources of new knowledge and deliberate decision-making (Martin-Breen and Anderies, 2011; Šūmane et al., 2018). Facilitating knowledge sharing within teams and enhancing information alignment can ensure that members’ capabilities match task requirements, thereby enabling the team to address complex problems in organizational environments (Hagemann and Kluge, 2017).

This paper aims to explore a new approach to understanding dynamic team functioning by examining the relationship between knowledge-creation-based team dynamic capabilities and team resilience. The serial mediation is conceptualized as a team behavioral mechanism, which facilitates the transition from knowledge creation to knowledge application for effective decision-making. It further reveals the internal mechanisms underlying the formation of functional teams and identifies critical boundary conditions.

## 2 Theoretical background and hypotheses

### 2.1 Dynamic capability theory

Dynamic capability focuses on “doing the right things”(Teece, 2015), aiming to enable organizations to renew their capabilities to adapt to changing environment (e.g., exploration) (Dixon et al., 2014). As one of the dominant theories in the field strategic management in recent years (Di Stefano et al., 2014; Forkmann et al., 2018), dynamic capability theory is applied to organizations to explain how the frequency of effectively reconfiguring resources contributes to achieving competitive advantage (Braganza et al., 2017).

In various studies, the definition of dynamic capabilities differs depending on the purpose (Barreto, 2010). However, a widely accepted composite definition by key contributors to the dynamic capabilities literature is that dynamic capabilities are the abilities of an organization to purposefully create, extend, or modify its resource base (Helfat et al., 2009). Here, the “resource base” refers to the organization’s resources, including tangible and intangible assets, human capital, and capabilities. This definition is currently regarded by key contributors as the most comprehensive reflection of the dynamic nature of dynamic capability (Munoz-Penas et al., 2024). In stable environments (Helfat and Winter, 2011), dynamic capability research claims to explain continuous, routine-based organizational change (Wenzel et al., 2021). Specifically, dynamic capability is considered to be a potential for systematically solve problems, which benefits from its tendency to sense opportunities and threats, this enables organizations to make timely decisions and effectively implement strategic decisions and changes, and thereby ensuring the right direction (Ferreira et al., 2020).

In contemporary organizational and managerial processes undergoing change, the primary task is to identify the need for or opportunity of transformation (Helfat et al., 2009). Accordingly, dynamic capabilities are viewed as “a combination of broad organizational capabilities and specific actions that work together to achieve organizational transformation” (Yeow et al., 2018), as well as “a subset of organizational and individual capabilities specifically dedicated to strategic change” (Helfat and Raubitschek, 2018). Moreover, since dynamic capabilities often rely on three general learning processes—experience accumulation, knowledge articulation, and knowledge codification (Zollo and Winter, 2002)—to enable capability evolution within organizations, it can be argued that knowledge constitutes the cognitive foundation of dynamic capabilities.

As in most of literature on focuses on the ability of an organization to sense, seize, and transform its ecosystem (Teece, 2007; Teece, 2014). This three-dimensional categorization enhances the conceptual consistency and clarity of dynamic capabilities (Eriksson, 2014). Dynamic capabilities, as higher-order capabilities (Cepeda and Vera, 2007; Collis, 1994; Teece, 2023; Winter, 2003), support the internal development of necessary competences (Gulbrandsen et al., 2017), thereby enabling unconventional managerial decision-making to play a greater role. In the higher education sector, institutions have been increasingly challenged for their capacity to respond to technological advancements, rapid commercialization, and social change (Bejinaru, 2017). The application of dynamic capabilities to higher education institutions is thus primarily reflected in their responsibility to function as innovative ecosystems that create value for society (Faccin et al., 2022). Dynamic capabilities are closely linked to innovation capacities in higher education (Akram and Hilman, 2017), and this theoretical lens offers a structural approach for strategic management within such institutions (Teece, 2018). It also assists in analyzing institutional activities in complex contexts (Heaton et al., 2019), with the aim of maximizing internal knowledge flow (Heaton et al., 2019; Kelleher and Ulrichsen, 2023).

### 2.2 Team dynamic capability

In knowledge-intensive domains, dynamic capabilities are essentially the development of knowledge-based dynamic capabilities (Kaur, 2019). Logically, dynamic capabilities are founded on all human-related factors, including skills, shared practical knowledge, and knowledge that facilitates purposeful action, indicating that knowledge creation serves as the operational pathway through which dynamic capabilities are actualized (Eisenhardt and Martin, 2000). Through knowledge creation, teams are able to establish new routines (Dong et al., 2004; Li and Chen, 2024). However, current research has primarily emphasized the “*adaptive”* dimension of dynamic capabilities—namely, the rapid reconfiguration of existing knowledge to respond to environmental changes. Much of this scholarship focuses on how teams develop dynamic capabilities to enhance firm performance, such as team learning capabilities (Harvey et al., 2018) and team R&D capabilities (Wang and Luo, 2013), or capabilities related to resource absorption, integration, decision optimization, and trust-based communication (Zhang et al., 2017). In contrast, relatively limited attention has been given to the “*creative”* dimension of dynamic capabilities—namely, the capacity to generate new knowledge and respond to complex environments.

High-quality collaboration for problem-solving requires the sharing of resources and skills among team members (Mola et al., 2020), enabling problem resolution through processes that foster knowledge renewal and collaboration. Ambrosini and Bowman (2009) argued that dynamic capabilities emerge from the co-evolution of tacit experiential accumulation and the articulation and codification of explicit knowledge activities. From the perspective of internal organizational concretization processes (Eisenhardt and Martin, 2000), creative dynamic capabilities are rooted in meso-level team activities within organizations—that is, in the interactions among team members (Nonaka et al., 2016). Nonaka et al. (2016) proposed a complementary approach integrating the SECI model (socialization, externalization, combination, internalization) with the dynamic capabilities framework, emphasizing that the knowledge creation process serves as the operational pathway of team dynamic capabilities. In other words, the knowledge creation process forms the foundation of team dynamic capabilities. Given the significant role of knowledge sharing in enhancing team effectiveness (Alsharo et al., 2017; Kim et al., 2023; Lin and Huang, 2020), dynamic knowledge creation is likely to constitute a form of team dynamic capability. That is, when team members possess complementary expertise and have mutual awareness of one another’s knowledge and tasks, teams are more capable of dynamic functioning (Salvato and Vassolo, 2018), thereby enabling the realization of the true value of team collaboration (Thayer et al., 2018).

Previous literature has largely acknowledged that the knowledge evolution phase of dynamic capabilities emphasizes both cognitive and behavioral foundations simultaneously (Dong et al., 2004; Nonaka et al., 2000). The evolutionary process of knowledge-based dynamic capabilities is characterized by a cyclical progression through four stages—variation, internal selection, retention, and propagation—indicating that cognitive efforts and behavioral efforts occur concurrently (Dong et al., 2004). Leadership, as a behavioral foundation of dynamic capabilities (Gheitarani et al., 2023), was integrated early on into the dynamic knowledge creation process through the unified model of SECI, Ba, and leadership (Nonaka et al., 2000). Similarly, the integrated model of knowledge-based dynamic capabilities (Nonaka et al., 2016) presents a unified process encompassing both cognitive and behavioral bases.

However, the value and impact of dynamic capabilities at the team level remain ambiguous and contested (Schilke, 2014). Wilden et al. (2016) argued that the research on dynamic capabilities should shift from the question of whether they influence performance to how they do so. Existing literature confirms that the mechanism through which dynamic capabilities lead to performance outcomes remains an unresolved issue in empirical research (Eriksson, 2014; Zhou et al., 2019). In other words, the “how” question has yet to be fully addressed (Wenzel et al., 2021). In light of this, recent research has begun to explore the indirect effects of dynamic capabilities (Baía and Ferreira, 2019; Laaksonen and Peltoniemi, 2018).

As previously noted, the dynamic knowledge creation process (SECI) inherently encompasses both cognitive and behavioral effort components. However, as Nonaka and Takeuchi (2021) emphasized, while knowledge creation enables teams to acquire new insights, it does not necessarily endow them with the wisdom to apply that knowledge effectively. The true value of knowledge must be realized through “knowledge practice.” According to Duchek (2020), resilience depends on the interplay between cognitive and behavioral capabilities and concrete actions. Building on the Knowledge-to-Action (KTA) Framework proposed by Graham et al. (2006), it is argued that following knowledge creation, teams members must apply, utilize, and translate knowledge into action in order to achieve resilience.

### 2.3 Team resilience

Teams are essentially goal-oriented entities, and team resilience is recognized as the team’s ability to handle problems and overcome obstacles (Alliger et al., 2015; Lengnick-Hall et al., 2011). This capability enables teams to proactively adjust their course of action to successfully execute specific tasks, thereby enhancing reliability, sustainability, and overall performance (Hartwig et al., 2020; Marchese et al., 2018; Specking et al., 2019). Theoretically, resilient teams are capable of rapidly identifying, designing, and implementing change while avoiding new obstacles that may impair team effectiveness beyond their current capabilities (Hoffman and Hancock, 2017). Given that interpersonal relationships form the foundation of resilience (Luthar, 2015), team resilience has been described as an emergent state arising from shared beliefs among team members (Kennedy et al., 2016), a collective outcome (Morgan et al., 2017), and a team goal cultivated through a shared vision, mutually agreed-upon “team charter,” and regular reinforcement (Morgan et al., 2019). On this basis, scholars have called for exploring team resilience through a consensus-based perspective that emphasizes emergence from team member interactions (Chapman et al., 2020; Raetze et al., 2021). This interpretation aligns with prior research that uses individual-level data to study collective phenomena when aggregation is not feasible.

Given its inherently social nature, a major research direction in team resilience focuses on team resources and interaction processes. For instance, team efficacy and collaboration (Strode et al., 2022), team personality (Mitchell et al., 2025), emotional and behavioral integration (Chen and Zhang, 2021; Hartmann et al., 2021), and the dynamic management of knowledge differences (Liu et al., 2023) have all been identified as factors that can foster team resilience (Liu and Harms, 2025). These research trends align with the recommendation in the dynamic capabilities literature to integrate organizational theories (e.g., resilience) and systems thinking to explore the development and functioning of dynamic capabilities in broader contexts (Arndt, 2019). From a knowledge management perspective, previous studies have demonstrated a significant positive correlation between knowledge management and resilience (Ibrahim Ismael et al., 2021; Singh et al., 2025; Yao et al., 2025). Particularly in certain professional environments, maintaining resilience at one level may require transformative change at another. In this sense, resilience is largely about organizational members learning how to change in order not to be changed (Walker, 2020), especially given the critical importance of knowledge integration within higher education institutions (Zahra et al., 2020).

### 2.4 Communicative

Communication encompasses all processes by which one person’s perspective influences another’s; it is a social behavior used to share attitudes, viewpoints, information, knowledge, and opinions (Shannon and Weaver, 1949). In organizational management, communication refers to the comprehensive process of sending, receiving, and exchanging multi-level and multi-content information among organizational members. It serves as the foundation for information transmission between individuals, institutions, and organizations, reflecting the processes of organizational design, management, and implementation. Therefore, it is critical to planning and decision-making (Khan et al., 2017). Particularly in the context of strategic change, communication plays a key role in how organizations deliberately use it to fulfill their overall mission (Frandsen and Johansen, 2017; Hallahan et al., 2007).

In knowledge management, the acquisition, transfer, and utilization of knowledge constitute the systemic flow of knowledge resources (Drucker, 1988). Communication is therefore regarded as one of the integral components of the dynamic knowledge evolution process, as exemplified in the SECI model (Nonaka et al., 1996). In this process, communication primarily functions to establish new routines for knowledge through widespread dissemination within the organization. Based on team collaboration (Gervits et al., 2016), communicative emphasizes collaboration among team members grounded in communication to facilitate knowledge creation. For instance, task-oriented, process-oriented, and socio-emotional-oriented communication allow team members to alternately focus on task demands, coordination issues, and interpersonal relationships, respectively (Stempfle and Badke-Schaub, 2002). Although these three types of communication differ significantly in their effectiveness for problem solving (Stempfle and Badke-Schaub, 2002), open communication channels within teams can still foster shared understanding through dialogic interaction (Clark and Schaefer, 1989).

However, as new knowledge accumulates into knowledge assets, its practical application often encounters common obstacles. Team members’ limited understanding of newly generated knowledge may hinder its enactment. In other words, the transition from knowledge creation to implementation is inherently a socialized process dependent on relational interactions; thus, dissemination and communication are essential for translating knowledge into action (Gagnon, 2011). According to sensemaking theory (Weick, 1995), sensemaking is viewed as a set of mechanisms that organizations typically use to define norms and rules for perceiving, interpreting, trusting, and acting (Sackmann, 1991). Communicative functions as one such behavioral mechanism, aiming to facilitate strategic information exchange among team members and serve as a foundation for decision-making processes.

### 2.5 Leadership

Leadership is characterized as an influential relationship among [people] aimed at achieving genuine transformation that reflects shared goals (Rost, 1993). It is regarded as a collective phenomenon shared among employees (Denis et al., 2012; Lord et al., 2017; Will, 2016; Yammarino et al., 2012) and a social phenomenon (Mumford et al., 2012), indicating that the focus of leadership development has shifted from individuals to the cultivation of relationships, teams, networks, and organizations (Day, 2000; Stogdill and Shartle, 1948). In other words, leadership is founded on social capital, transitioning from leader competencies to dialogic relationships (Eva et al., 2021). Simply put, leadership is not about a single individual or a position; rather, it represents a complex moral relationship between individuals, founded on trust, obligation, commitment, emotions, and a shared noble vision (Ciulla, 2004). It is an emergent attribute of interactions aimed at collective outcomes (Denis et al., 2012).

Nonaka and Toyama (2002) emphasize that the integrative function of leadership lies in “creating a knowledge vision, managing the knowledge-creation space, maintaining creative routines, and building an incentive system.” In other words, effective knowledge creation requires a pragmatic and dialogical form of leadership (Nonaka and Toyama, 2007). Leadership research has linked specific management styles to successful knowledge management (Lakshman, 2009). For example, empowering leadership is positively associated with knowledge management practices (Singh, 2008; Türkmendağ and Tuna, 2022), and transformational, transactional, knowledge-oriented, and strategic leadership styles have all been found to exert sustained and positive influences on knowledge management processes (Al Amiri et al., 2020). At the team level, distributed leadership is also recognized as a vital source of dynamic creativity (Nonaka et al., 2016). Some studies specifically highlight the critical role of leadership in knowledge management activities and processes—for instance, Von Krogh et al. (2012) integrated concepts such as Ba (knowledge-creating context), the SECI model, knowledge assets, and leadership behaviors to develop a knowledge-creating situational leadership framework (Nonaka and Konno, 1998; Nonaka et al., 1996), as well as the unified model of dynamic knowledge creation proposed by Nonaka et al. (2000).

In certain transformational environments, teams are primarily oriented toward problem-solving capabilities. Sternberg et al. (2021) suggest that employing the “meta-intelligence” model enables flexibility in generating problem-solving strategies. For instance, leaders may first use their creative skills to generate novel and high-quality solutions; second, they apply analytical skills to evaluate the logical coherence and feasibility of the proposed solutions; and third, they utilize wisdom-based skills to ensure that solutions contribute to the common good (Sternberg, 1998; Sternberg, 2003a; Sternberg, 2019; Sternberg, 2020). Therefore, leadership should influence the generation, sharing, and application of knowledge (Mac Gillivray, 2018). From a knowledge-practice perspective, effective leadership plays a critical role in decision-making by developing and integrating three core skills—wisdom, intelligence, and creativity (WICS) (Sternberg, 2003b). For example, knowledge-based leadership has been shown to encourage the development and application of knowledge practices (Donate and de Pablo, 2015). Given the practical challenges in knowledge management (Ding et al., 2019) and the value of dynamic capabilities as a basis for innovation in strategic planning (Nonaka et al., 2016), wise leadership consistently aims to guide team members toward decisions that benefit the collective (Nonaka and Takeuchi, 2011). Leaders who possess practical wisdom can optimistically enhance a team’s dynamic capabilities (Nonaka et al., 2016). Therefore, wise leadership teams may actively contribute to fostering environments conducive to knowledge sharing, encouraging member participation, and enhancing team spirit, thereby improving the efficiency of knowledge practices (Ding et al., 2019).

### 2.6 Interpretation

The response of a team to problems is partly based on how these issues are understood and interpreted (Ashmos et al., 1998; Burke et al., 2018; Dutton and Jackson, 1987; Gagné, 2018; Hitt and Tyler, 1991; Schneider and De Meyer, 1991; Thomas et al., 1993; Thomas and McDaniel, 1990; Von Krogh, 2018). Interpretation is an expression of collective thought (Weick and Roberts, 1993), the team needs to develop a shared processing mechanism to interpret events (Walsh and Ungson, 1991).

In strategic management, information is utilized to interpret environmental changes, generate new knowledge, and inform decision-making on courses of action. These processes involve interwoven behaviors that contribute to the organization’s deep interpretation of information (Choo, 1996). Prior studies have examined how decision-makers interpret strategic events through the lens of strategic issue diagnosis (SID) (Dutton et al., 1983). The SID framework emphasizes the need to construct understanding and assign meaning to an issue before linking it with potential solutions, thereby helping decision-makers avoid the “blindness” effect in strategic sensemaking (Dutton et al., 1983). Generally, interpretation is defined as an organization’s ability to make sense of its environment in multidimensional ways, especially under conditions of strategic complexity (Neill et al., 2007). This involves constructing cognitive frames to encode environmental cues and assign meaning to them for situational understanding. In a team context, interpretation focuses on how team members use the information and knowledge they possess to influence others (Thomas and McDaniel, 1990) and shape decision rationales. When new knowledge encounters barriers, teams may need to engage in reconstructive interpretation (Franke, 2023) and adopt an interpretive turn (Nicklin, 2012) to explore dynamic and decentralized frameworks for knowledge practice. This requires attention to how purposeful interpretive spaces are created (Liedtka, 2000), and how such interpretations either facilitate or inhibit collective team action within the broader knowledge system.

### 2.7 Analytical

Analytical is defined as “judgment based on psychological reasoning” (Allinson and Hayes, 1996) and refers to a rational, thoughtful, and conscious judgment (Epstein et al., 1996). It represents the extent to which data, facts, and logical reasoning are relied upon when addressing problems, wise decision making, or conducting evaluations (Wang et al., 2017). In the context of knowledge creation, team members primarily integrate diverse forms of both tacit and explicit knowledge to generate innovative ideas (Olaisen and Revang, 2018; Von Krogh et al., 2000). The analytical mode of knowledge creation refers to the understanding and interpretation of characteristics of the (natural) world (Moodysson et al., 2008). Previous research has shown that predictive analytics can influence knowledge creation in marketing (Hair, 2007). The decision-making process itself enhances the understanding of both the issue at hand and the processes involved. In other words, decision-making and knowledge creation are interdependent processes.

However, team members primarily apply new knowledge for problem-solving purposes. Previous research suggests that many problem-solving approaches are analytically driven and supported (Hoerl and Vining, 2021). Analytical capability, as a core component of the metacognitive approach to problem-solving (Sternberg et al., 2021), can be examined through the WICS framework (wisdom-intelligence-creativity-synthesized) (Sternberg, 2003a; Sternberg, 2019), which emphasizes the interaction among creative, analytical, and practical mechanisms in shaping a team’s ability to solve problems effectively. In particular, multi-perspective analysis reflects the team’s sensemaking capacity, requiring the ability to integrate diverse viewpoints, perform cognitive blending in decision-making processes, and transform problem identification into deep understanding (Neill et al., 2007). From this perspective, broad and diverse new knowledge indeed supports sustainable action processes and capacity building within teams (Caniglia et al., 2021).

### 2.8 Research gaps and positioning

As noted earlier, the dynamic knowledge creation process (SECI) already involves both cognitive and behavioral efforts. However, following Wilden et al. (2016), we argue that the research focus on dynamic capability literature should shift from whether it affects performance to how it does so. Our position is that existing literature has not fully answered the “how” question (Wenzel et al., 2021). Although prior research has addressed the “*adaptive*” dimension of dynamic capability, few have examined its “*creative*” dimension and subsequent knowledge enactment. As noted by Nonaka and Takeuchi (2021), knowledge only realizes its true value when it is enacted through purposeful practice. Based on the above-identified research gaps, the novelty of this study lies in drawing on the concept of Knowledge-to-Action (KTA) to explain how team dynamic capability contributes to team resilience.

In this paper, the theoretical foundation of the mediating mechanisms is rooted in sensemaking theory, particularly the three core dimensions widely recognized in organizational literature—communicative, interpretation, and analytical (Weick, 1995; Neill et al., 2007). These constructs have often been studied as dimensions of sensemaking capability. Building on this foundation, we intentionally disaggregate these three dimensions to examine their individual mediating roles. Moreover, we introduce wise leadership (Nonaka et al., 2016)—a construct closely linked to knowledge creation—as an additional and interrelated mediator. This combination forms a set of related yet distinct mediating mechanisms, which reflects a new theoretical perspective: it explains how team dynamic capability contributes to team resilience through the functional integration of sensemaking and knowledge activation processes. However, these four constructs are not dimensions of team dynamic capability, Instead, they are functional enabling mechanisms that emerge following the team’s dynamic knowledge creation process. In other words, the newly created knowledge enabled by dynamic capability requires knowledge enactment—through communicative, leadership, interpretation, and analytical—in order to generate value and inform resilient decision-making.

### 2.9 Hypothesis development and theoretical framework

#### 2.9.1 Team dynamic capability and communicative

In environments characterized by change and innovation, collaboration and dynamic capability are often integrated as fundamental conditions for organizations. This implies that all participants need to contribute resources and expertise as complementary assets and capabilities to jointly bear costs and risks for a common goal (Dodgson, 2014). The creativity of dynamic capability arises from team-level interactions among members (Nonaka et al., 2016). Once new knowledge is created, team members must determine which knowledge to apply based on the specific contextual environment. This process inevitably requires information exchange to clarify each other’s capabilities and intentions in order to formulate strategies and plan tasks. Strategic communication, in particular, involves members purposefully engaging in dialogs that are meaningful to their collective objectives, playing a unique role throughout the processes of strategy formulation, revision, articulation, execution, implementation, and operation (Zerfass et al., 2020). Teams construct meaning for newly generated knowledge through communication, enabling members to build shared understanding (Hallahan et al., 2007) and reach decisions that satisfy multiple stakeholders. Thus, newly created knowledge within teams facilitates strategic information exchange among members. Therefore, this paper proposed that.

*H1*: Team dynamic capability positively affects communicative.

#### 2.9.2 Team dynamic capability and leadership

Team dynamic capability primarily aims to build team efficiency in order to generate team performance. As a component of team collaboration quality, the knowledge creation process represents a “creative” form of team dynamic capability and constitutes the innovative value underlying organizational development planning (Nonaka et al., 2016). It requires leadership that integrates “pragmatism and dialog,” such as articulating a knowledge vision, managing “Ba” (shared spaces), maintaining innovation trajectories, and establishing incentive systems (Nonaka and Takeuchi, 2007). Therefore, it can be regarded as a facilitating condition for the enactment of team leadership functions (Bradshaw et al., 2015). Since the knowledge creation process is grounded in interpersonal interaction and engagement among team members, this team-rooted interaction pattern helps foster pragmatic and wise leadership (Nonaka et al., 2016). Conversely, wise leadership characterized by a “middle-up-down” approach allows team members to sense changes, seize opportunities, and enhance their management awareness. It cultivates a collective consciousness of participatory governance, in which every member perceives themselves as contributing to team management (Adler and Hiromoto, 2012), thereby providing momentum for knowledge enactment. Therefore, this paper proposed that.

*H2*: Team dynamic capability positively affects leadership.

#### 2.9.3 Team dynamic capability and interpretation

As a team dynamic capability, the output of knowledge creation is the generation of rich new ideas, representing the realization of novel concepts (Nonaka et al., 1994). However, whether newly created knowledge can be effectively applied to the environment depends on the team’s ability to interpret external complexity, thereby enhancing the alignment between new knowledge and environmental demands. This interpretive process occurs within the shared context of “Ba” established by the team (Nonaka et al., 2001). The “Ba” can be understood both as a supportive environment and as an interpretive tool (Al-Mansour and Obembe, 2021). The energy generated within “Ba”—namely, the team members’ interpretation—can significantly enhance its effectiveness. In other words, the practical application of new knowledge requires active interaction among participants within various forms of “Ba,” guided by the team’s shared vision. The interaction and communication processes that take place in this shared environment constitute both interpretive and integrative mechanisms of information and knowledge. Learning and sharing activities help strengthen team members’ absorptive capacity, beliefs, and values, which in turn facilitate the interpretation of information (Abbas et al., 2020). Put differently, the practice of newly created knowledge allows team members to recognize how the team’s interpretive function contributes to overall team performance. Therefore, this paper proposed that.

*H3*: Team dynamic capability positively affects interpretation.

#### 2.9.4 Team dynamic capability and analytical

The knowledge creation process is a systematized management activity that requires deliberate facilitation. When conceptualized as a team dynamic capability, knowledge creation implies that the new knowledge generated is rich in data and information, serving as essential input for analytical processes. Analytical is inherently a multiple perspective consideration (Neill et al., 2007), requiring team members to engage in diverse viewpoints when evaluating the application of new knowledge. The goal is to generate more contextualized insights and integrate them into coherent solutions, which plays a critical role in team-based problem solving (Srivastava et al., 2006; Zimmermann et al., 2021). Moreover, analytical within teams is often conducted through multiple-criteria decision-making processes, wherein team members evaluate alternative courses of action based on various competing priorities (Belton and Stewart, 2012). In this regard, newly created knowledge can promote rational, integrative thinking among team members, enabling more effective and structured decision-making processes in response to complex problems. Therefore, this paper proposed that.

*H4*: Team dynamic capability positively affects analytical.

#### 2.9.5 Communicative and team resilience

Previous research has shown that team communicative actions can legitimize new knowledge, learning, and practices (Del Corso et al., 2015). From a problem-solving perspective, team communication behaviors enhance resilience by leveraging new knowledge to explore problem domains (Vos, 2017) and by facilitating communication-centered coordination (Doerfel et al., 2022). Team resilience largely depends on the existence and quality of interpersonal relationships (Carmeli et al., 2021). High-quality connections (HQCs) are generative and vital (Aarrestad et al., 2015) and are particularly valuable for resilience (Stephens et al., 2013), serving as an important resource for the formation of team resilience capabilities (Carmeli et al., 2013; Stephens et al., 2013). That is, team members who engage in proactive and communicative interactions can cultivate a high-quality communicative atmosphere. This active relational process can facilitate (or hinder) information sharing, learning processes, and the development of adaptive problem-solving solutions (Paulus and Nijstad, 2003). If members perceive that their team relationships contribute to generating new ideas and identifying new opportunities, they tend to be more resilient (Carmeli et al., 2013). However, since the strength of relationships among team members is measured by the duration or length of the relationship and the frequency of communication (Granovetter, 1973). Normative communication patterns, such as transparent team communication, can enhance trust among members and increase the value of members’ willingness to disclose information (Lee and Li, 2020). Through generating new ideas and establishing consistency within the team, team communication reflects its importance in shaping team resilience (Carmeli et al., 2013; Gomes et al., 2014). Based on the value of open and trusting team communication, a positive voice atmosphere has the potential to influence team resilience (Brykman and King, 2021). Therefore, this paper proposed that.

*H5*: Communicative positively affects team resilience.

#### 2.9.6 Leadership and team resilience

Leaders play multifaceted roles in fostering resilience within organizations (McEwen, 2022), such as consistently demonstrating support for the team’s mission (Southwick et al., 2017). Literature has established that leadership (Bowers et al., 2017; Çop et al., 2021; Williams et al., 2017) is a key organizational factor influencing resilience and sustainability. Leaders can provide guidance and support to team members to effectively manage any disruptions (e.g., stress; Harms et al., 2017).

Leaders must guide the team in achieving its goals, encourage employee development, and instill a sense of participation and commitment (Lombardi et al., 2021). However, leaders must acquire practical wisdom or phronesis to enable team members to produce experiential knowledge that aligns with ethical standards (Nonaka and Takeuchi, 2011). Based on practical wisdom is vital for organizational resilience, effective communication, and societal well-being in complex governance (Awashreh and Hamid, 2024), it can be argued that teams characterized by wise leadership are more likely to enhance their resilience and strength. Therefore, this paper proposed that.

*H6*: Leadership positively affects team resilience.

#### 2.9.7 Interpretation and team resilience

The ability to solve problems largely depends on whether team members can effectively utilize the knowledge resources available within the team (Lin et al., 2015). Team members perceive stimuli from the environment, categorize information into frameworks or update reference frameworks, communicate with one another, and then adopt the most favorable approach. This team process occurs through a simultaneous engagement of belief and skepticism regarding the interpretation of specific situations (Talat and Riaz, 2020).

From a sensemaking perspective, effective team management and coordination facilitate the interpretation of current situations (Klein et al., 2010), helping team members understand unexpected events or unforeseen stimuli they encounter (Maitlis and Christianson, 2014). As a sensemaking capability, interpretation also represents the extent to which teams absorb and understand external environmental conditions and information (Neill et al., 2007). It represents the team’s capacity to perceive environmental conditions and to form a collective understanding of external situations. Through this interpretive process, the absorption of information enables teams to identify a wider range of opportunities and to leverage knowledge more effectively, thereby gaining advantages that contribute to sustained resilience (Yuan et al., 2022). Therefore, this paper proposed that.

*H7*: Interpretation positively affects team resilience.

#### 2.9.8 Analytical and team resilience

Information processing at the team level is, to varying degrees, thoughtful, effortful, and systematic (De Dreu and Beersma, 2010). Systematic information processing is typically characterized by open and thorough information exchange and the diligent integration of information (De Dreu, 2007). However, the complexity of problems encountered in many professional workplaces often exceeds the cognitive capacity of any single individual. Therefore, it is necessary for teams to develop shared knowledge (Bierhals et al., 2007) and build high-quality relationships among team members (Carmeli et al., 2021). These factors reflect a team’s collective problem-solving capacity (Hung, 2013), as evidenced by team’s analytical thinking, decision-making capability, root cause analysis, and reflective learning. Additionally, the degree of team cognition serves as the foundation for solving complex problems (Hung, 2013). As a psychological state that allows team members to anticipate and coordinate (Niler et al., 2021), diversity in team cognition can foster psychological safety within the team, thereby enhancing resilience (Paschertz, 2021). Therefore, this paper proposed that.

*H8*: Analytical positively affects team resilience.

#### 2.9.9 Communicative as a mediator

Communication has long been regarded as a vital tool for team collaboration and coordination (Mintzberg, 1973) because it encourages information exchange and knowledge sharing (Dzamtoska-Zdravkovska and Haque, 2023; Malik et al., 2024). In team contexts, the outcomes of individual learning are often transmitted to other members through socio-psychological mechanisms such as communication, collaboration, and proactive information sharing (Carmeli et al., 2021). Communication is widely recognized as a crucial tool for problem-solving (Bodunde et al., 2024; Clark et al., 1994), as it facilitates deeper information processing, encourages the exchange of unique insights, and serves important socio-emotional functions within the team (Mesmer-Magnus and DeChurch, 2009). In particular, when team members maintain high-quality relationships and engage in high-quality communication, they are more likely to generate creative solutions to problems (Carmeli et al., 2021). This is because the strength of team connections depends on the collective knowledge, skills, and attitudes possessed by the team (Hartmann et al., 2020).

Research indicates that a key attribute of resilient teams is their respect for professional expertise (Alliger et al., 2015), which implies that resilient team members tend to rapidly exchange available information. In this regard, the dynamic capability of knowledge creation within teams facilitates the development of high-quality connections, thereby strengthening team resilience (Hartwig et al., 2020). Conversely, a lack of resilience hinders the establishment of high-quality connections and undermines the creation of a constructive communicative climate (Brykman and King, 2021). Taken together, the connectivity among team members may serve as a critical mechanism that enables teams to identify opportunities amid challenges and generate novel insights, thus enhancing team resilience and making the team more resourceful (Carmeli et al., 2013). Therefore, this paper proposed that.

*H9*: Communicative mediates the relationship between team dynamic capability and team resilience.

#### 2.9.10 Leadership as a mediator

Previous literature has widely acknowledged that leaders play a pivotal role in the evolution of dynamic capabilities within organizations (Salvato, 2003), serving as microfoundations of such capabilities (Burton and Dickinger, 2025). The dynamic capability of knowledge creation emphasizes a condition in which participants engage in spontaneous collaboration and shift between roles of leadership and followership (Von Krogh et al., 2012), suggesting that leadership is distributed among team members. It is viewed as a collective attribute and a set of functions that the team must collectively fulfill (Gibb, 1954). This implies that, in the practice of knowledge, team members are expected to jointly assume leadership roles.

At the same time, the development of wise leadership is grounded in knowledge (Hassi and Storti, 2023), whereby new knowledge must be contextualized and integrated with real-world situations to generate team wisdom. This entails making judgments and taking actions in the process of applying knowledge and fostering a reflective and dialogical environment to cultivate practical wisdom. As one of the critical antecedents of team resilience (Hartwig et al., 2020), wise leadership may thus serve as an important mediating mechanism between dynamic capability and team resilience. Therefore, this paper proposed that.

*H10*: Leadership mediates the relationship between team dynamic capability and team resilience.

#### 2.9.11 Interpretation as a mediator

Interpretation is a meaning-making ability that teams employ to handle complexity (Neill et al., 2007). Within the dynamic capabilities framework, team members must be adept at identifying and evaluating internal and external informational cues in order to translate knowledge into coherent action. The purpose of interpretation is to enhance the team’s decision-making quality. However, possessing new knowledge does not necessarily guarantee better decision outcomes (Stoverink et al., 2020). Team members often differ significantly in terms of roles, responsibilities, experience, and expertise, and there is frequently information asymmetry, with each individual holding distinct and unevenly distributed knowledge.

New knowledge is interpreted by team members based on their unique experiences and perspectives, a process that can result in multiple, and sometimes conflicting, interpretations. To form an accurate situational assessment, teams engage in sensemaking through the contributions of those with the most relevant expertise and practical experience (Butchibabu et al., 2016). ultimately guiding collective decisions. Research indicates that risk interpretations formed through the decoding of risk-related knowledge can enhance resilience regardless of environmental fluctuations (Odiase et al., 2020). Interpretation, as a dynamic team function, enables the transformation of individual cognition into organized responses, playing a crucial role in resilience under pressure. Therefore, this paper proposed that.

*H11*: Interpretation mediates the relationship between team dynamic capability and team resilience.

#### 2.9.12 Analytical as a mediator

Achieving team performance and effectively leveraging newly created internal knowledge through knowledge sharing and renewal is inherently a complex task, which requires a certain degree of decision-making capacity (Abubakar et al., 2019). Analytical is not only a rational and structured mode of information processing but also represents a multi-perspective approach to thinking (Neill et al., 2007), in sharp contrast to intuition-based judgment. It enables teams to critically evaluate knowledge, integrate diverse viewpoints, weigh multiple options, and make reasoned decisions—capabilities that are essential for navigating dynamic environments.

The knowledge creation process in teams typically involves integrating different perspectives through social interaction. This process not only enriches the team’s cognitive resources but also fosters analytical capability by encouraging systematic reasoning and reflective learning. When teams are able to understand problems from multiple angles, they can deepen their decision-making processes, thereby improving decision quality (Pacini and Epstein, 1999) and enhancing overall performance (Kaufmann et al., 2014; Smolka et al., 2018; Zhu et al., 2021). In environments characterized by constant change and uncertainty, external pressures significantly increase the complexity of decision-making. Teams must not only react swiftly but also respond wisely. As the decision-making process is closely linked to resilience (Mishra et al., 2015; Son et al., 2020), analytically grounded decisions are more likely to influence and enhance team resilience (Agassian, 2021). Therefore, this paper proposed that.

*H12*: Analytical mediates the relationship between team dynamic capability and team resilience.

**Figure 1:**
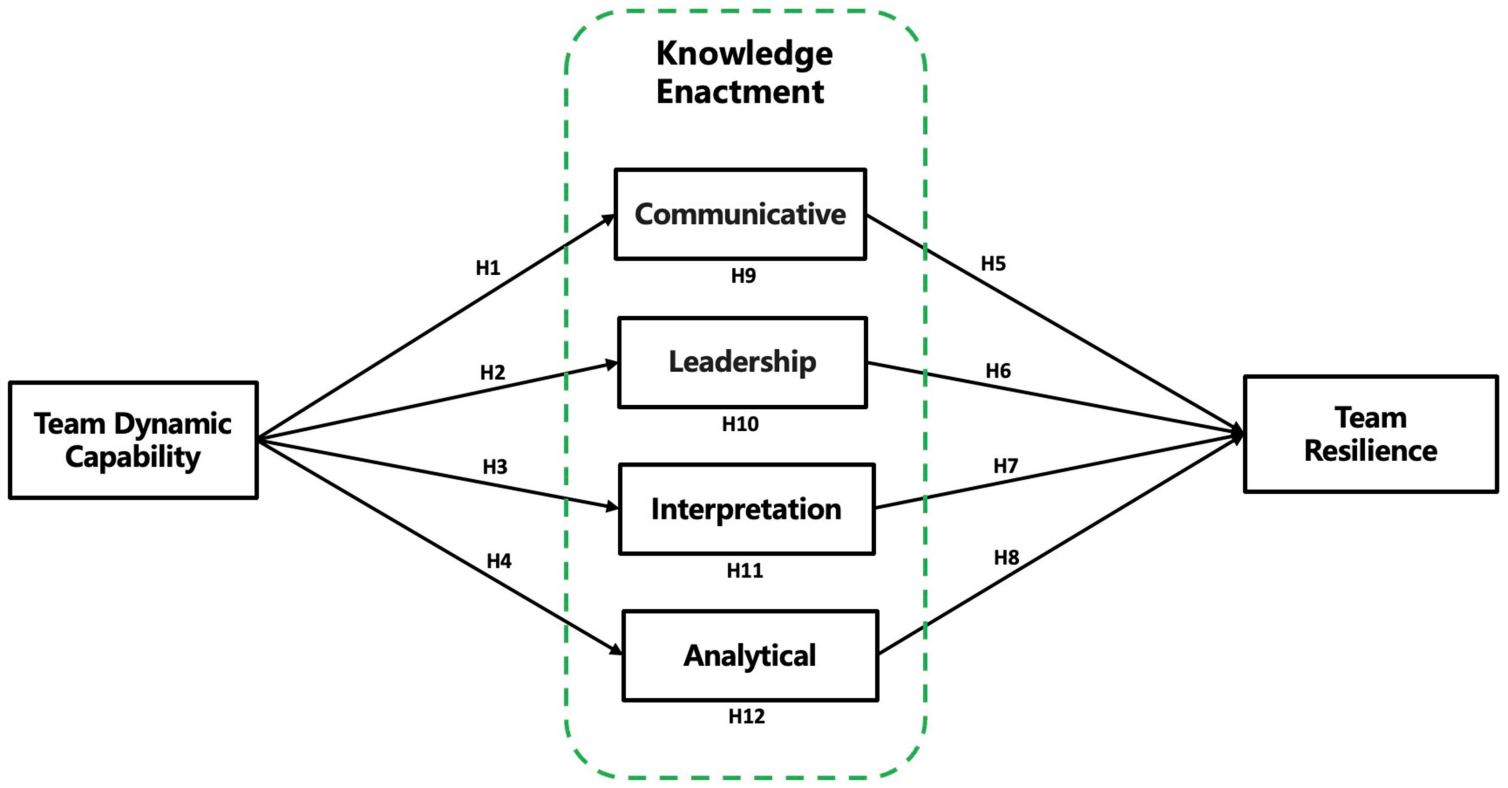
Conceptual model illustrating the mediating role of team competencies between dynamic capabilities and team resilience. Conceptual model illustrating how team dynamic capabilities influence team resilience through four functionally enabling mechanisms—communicative, leadership, interpretation, and analytical—collectively referred to as knowledge enactment.

In summary, this study conceptualizes the mediating mechanisms as functionally enabling mechanisms that emerge following the team’s dynamic knowledge creation process. These mechanisms are supported by team dynamic capabilities and serve as intermediaries between team dynamic capability and team resilience. Based on the development of the hypotheses, the conceptual model is constructed as shown in Figure 1.

## 3 Methods

Data collection was conducted primarily through an online survey (Tuten et al., 2002), which was designed in electronic format and distributed to participants via a hyperlink or QR code. Faculty and staff members from 64 public higher education institutions (e.g., academic or administrative professionals—given that Chinese public universities commonly adopt a dual-role system in which administrative staff also serve as academic personnel) were invited to complete the questionnaire. The survey was distributed across Guangxi Province through educational networks, allowing responses from a broad and diverse of public higher education institutions. Previous research has suggested that team resilience can be viewed as an isomorphic representation of individual resilience (West et al., 2009), as individuals within a team are typically directly or indirectly involved in all team-related activities. Accordingly, organizational or team-level resilience may be understood from the standpoint of employees, in a manner consistent with the transformational perspective (Kim, 2020). After distribution, collection, and screening in China, a valid sample size of 617 responses was obtained.

To operationalize the variables, a structured questionnaire was designed following a literature review and discussions on the variables. Team dynamic capability was conceptualized as a higher-order construct grounded in the SECI model, and measured using the KMSP-Q scale developed by Farnese et al. (2019). The scale comprises three dimensions—Socialization, Externalization and Combination, and Internalization—captured through 30 items. A second-order Confirmatory Factor Analysis (CFA) was conducted to empirically test the hierarchical structure of TDC, and results confirmed that all three dimensions significantly loaded onto the higher-order latent construct. However, to enhance model parsimony and ensure consistency with prior studies adopting the SECI framework (e.g., Nonaka et al., 2016), only the first-order CFA results were presented in the main analysis.

Team resilience was measured with 3 items adapted from Stephens et al. (2013). Communicative was measured with 4 items adapted from Akgün et al. (2014). Leadership was assessed using 6 items derived from Nonaka et al. (2016), as well as Ding et al. (2019). Interpretation was measured with 5 items adapted from Neill et al. (2007), while analytical was measured with 7 items adapted from Akgün et al. (2014). In total, the questionnaire consisted of 59 items, all assessed on a five-point Likert scale ranging from 1 (strongly disagree) to 5 (strongly agree).

Since the variable data from the scales in this study were derived from the same source of subjects, there may be an issue of common method bias (CMB). Therefore, two statistical control methods were employed to test for common method variance (CMV). First, Harman’s single-factor test was conducted to determine whether common variance was significant. This test involved factor analysis of all items the scale; if the variance explained by the first unrotated factor was less than 40% (Tang and Wen, 2020), it would indicate that common method bias was not severe. Second, this study further employed the effects of an unmeasured latent methods factor (ULMC) to test for CMV. In confirmatory factor analysis (CFA), the six-factor model of the core study served as the baseline model, incorporating the single method as a latent variable that was uncorrelated with the other factors in the six-factor model, allowing all test items to load on this latent variable. Finally, the differences in fit indices between the two models were compared. If the RMSEA and SRMR decreased by no more than 0.05, and the CFI and TLI increased by no more than 0.10, the model including the common factor would not show significant improvement over the model without the common factor, indicating that there was no serious issue of common method bias (Zhou and Long, 2004).

In terms of reliability, utilized the internal consistency coefficient method (Cronbach’s *α* > 0.70) and the split-half reliability coefficient (Spearman-Brown method) for reliability analysis. A split-half reliability coefficient exceeding 0.80 indicates good reliability (Wu, 2010). Validity analysis was primarily conducted via SPSS AMOS to construct a confirmatory measurement model to examine its convergent validity, discriminant validity, and construct validity (average variance extracted (AVE) exceeded 0.50), and composite reliability (CR) was greater than 0.70.

For hypothesis testing, a structural equation model (SEM) was constructed via SPSS AMOS to analyze the path relationships and assess the direct effects of team dynamic capability on communicative, leadership, interpretation, and analytical. The direct effects of communicative, leadership, interpretation, and analytical on team resilience, as well as the indirect effects of team dynamic capability on team resilience, were subsequently evaluated after the variables of communicative, leadership, interpretation, and analytical. The mediating effect was tested via the Sobel product coefficient method and the bootstrap method. The Sobel product coefficient method indicates the presence of a mediating effect if the Z value (absolute value) of the mediating effect is>1.96. The bootstrap sampling method (Hayes, 2009) was employed with both the percentile method and the bias-corrected percentile method to establish a 95% confidence interval. If the 95% confidence interval does not include 0 and *p* < 0.05, the indirect effect is significant, and the mediating effect is significant.

## 4 Results

A frequency analysis of the 617 valid sample responses revealed that there were 317 females (51.4%) and 300 males (48.6%). The sample population was predominantly aged centered between 31 and 40 age years (66.30%), with the highest number of qualifications holding a master’s degree (65.60%), followed by those with a doctoral degree (22.70%). This distribution reflects the common educational profile of professionals engaged in academic and administrative roles within public universities in China, where a master’s degree is typically the baseline qualification. Most participants had between 6 and 15 years of working experience (60.00%).

### 4.1 Common method variance

To Exploratory factor analysis of the scale variables revealed 8 factors with eigenvalues greater than1. The variance explained by the first unrotated factor was 32.815%, which is below the 40% threshold, indicating that there is no severe common method bias in the data of this study. After adding the common method latent factor, CMIN (*x*^2^) decreased by 44.092, degrees of freedom (*df*) decreased by 28, and CMIN/*df* (*x*^2^/*df*) decreased by 0.033. The RMSEA and SRMR values decreased by 0.002 and 0.003, respectively, whereas the CFI and TLI values increased by 0.002 and 0.001, respectively. The model fit did not change significantly, indicating that the CMV in this study’s data is not severe.

### 4.2 Reliability and validity analysis

#### 4.2.1 Reliability

The reliability results of the scales are shown in Table 1. All the concepts demonstrated reliability coefficients within the acceptable range, indicating good scale reliability.

**Table 1:** Results of reliability.

Concept	Indicators	Cronbach’s *α*	Spearman-Brown
1. Team Dynamic Capability (TDC)		0.956	0.860
	SC	0.927	0.929
	EC	0.917	0.916
	IT	0.930	0.928
2. Team Resilience (TR)		0.929	0.927
3. Communicative (CM)		0.864	0.861
4. Leadership (LD)		0.883	0.885
5. Interpretation (IN)		0.892	0.903
6. Analytical (AN)		0.919	0.914

#### 4.2.2 Validity

The convergent validity and composite reliability results for the overall scale variables are shown in Table 2. The AVE values for the 6 variables ranged between 0.557 to 0.813 (greater than0.5), and the standardized factor loadings for all the items were exceeded 0.50, indicating good convergent validity for each subscale. The composite reliability (CR) values for all subscales greater than 0.70, and the squared multiple correlations (SMC) for items all above 0.33, demonstrating good structural reliability.

**Table 2:** Results of convergent validity and composite reliability.

Concept	Item	Factor loading (Standardized)	Standard Error(SE)	Significant test(t)	SMC	AVE	CR
Team Dynamic Capability (TDC)	SC	0.756***	–	–	0.571	0.606	0.822
	EC	0.811***	0.052	18.208	0.658		
	IT	0.767***	0.049	17.543	0.588		
Team Resilience (TR)	TR01	0.900***	-	-	0.811	0.813	0.929
	TR02	0.906***	0.029	33.937	0.820		
	TR03	0.899***	0.029	33.460	0.809		
Communicative (CM)	CM01	0.848***	–	–	0.719	0.614	0.864
	CM02	0.723***	0.041	19.504	0.523		
	CM03	0.776***	0.042	21.381	0.602		
	CM04	0.781***	0.042	21.537	0.609		
Leadership (LD)	LD01	0.772***	–	–	0.596	0.557	0.883
	LD02	0.726***	0.054	18.232	0.527		
	LD03	0.729***	0.052	18.317	0.531		
	LD04	0.779***	0.054	19.770	0.608		
	LD05	0.750***	0.054	18.922	0.562		
	LD06	0.721***	0.052	18.098	0.520		
Interpretation (IN)	IN01	0.819***	–	–	0.671	0.623	0.892
	IN02	0.785***	0.046	21.581	0.616		
	IN03	0.793***	0.045	21.871	0.628		
	IN04	0.773***	0.045	21.146	0.597		
	IN05	0.777***	0.045	21.292	0.603		
Analytical (AN)	AN01	0.837***	–	–	0.701	0.618	0.919
	AN02	0.782***	0.038	22.755	0.612		
	AN03	0.795***	0.039	23.289	0.631		
	AN04	0.780***	0.039	22.670	0.609		
	AN05	0.776***	0.038	22.505	0.603		
	AN06	0.768***	0.038	22.170	0.590		
	AN07	0.762***	0.038	21.919	0.581		

The discriminant validity results for each scale are shown in Table 3. The AVE values for the six concepts ranged from 0.746 to 0.902, and the correlation coefficients among the concepts ranged from 0.333 to 0.579. The AVE values for each variable were greater than their corresponding correlation coefficients, indicating good discriminant validity among the six concepts. Therefore, the discriminant validity of the overall scale has been confirmed.

**Table 3:** Discriminant validity of the constructs.

Concept	TDC	TR	CM	LD	IN	AN
Team dynamic capability (TDC)	**0.778**					
Team resilience (TR)	0.579***	**0.902**				
Communicative (CM)	0.518***	0.557***	**0.784**			
Leadership (LD)	0.492***	0.544***	0.537***	**0.746**		
Interpretation (IN)	0.491***	0.558***	0.560***	0.428***	**0.789**	
Analytical (AN)	0.436***	0.600***	0.369***	0.333***	0.372***	**0.786**

The value of the diagonal (in bold): $\sqrt{\mathrm{AVE}}$ ; ***p < 0.001.

### 4.3 Hypothesis testing

**Figure 2:**
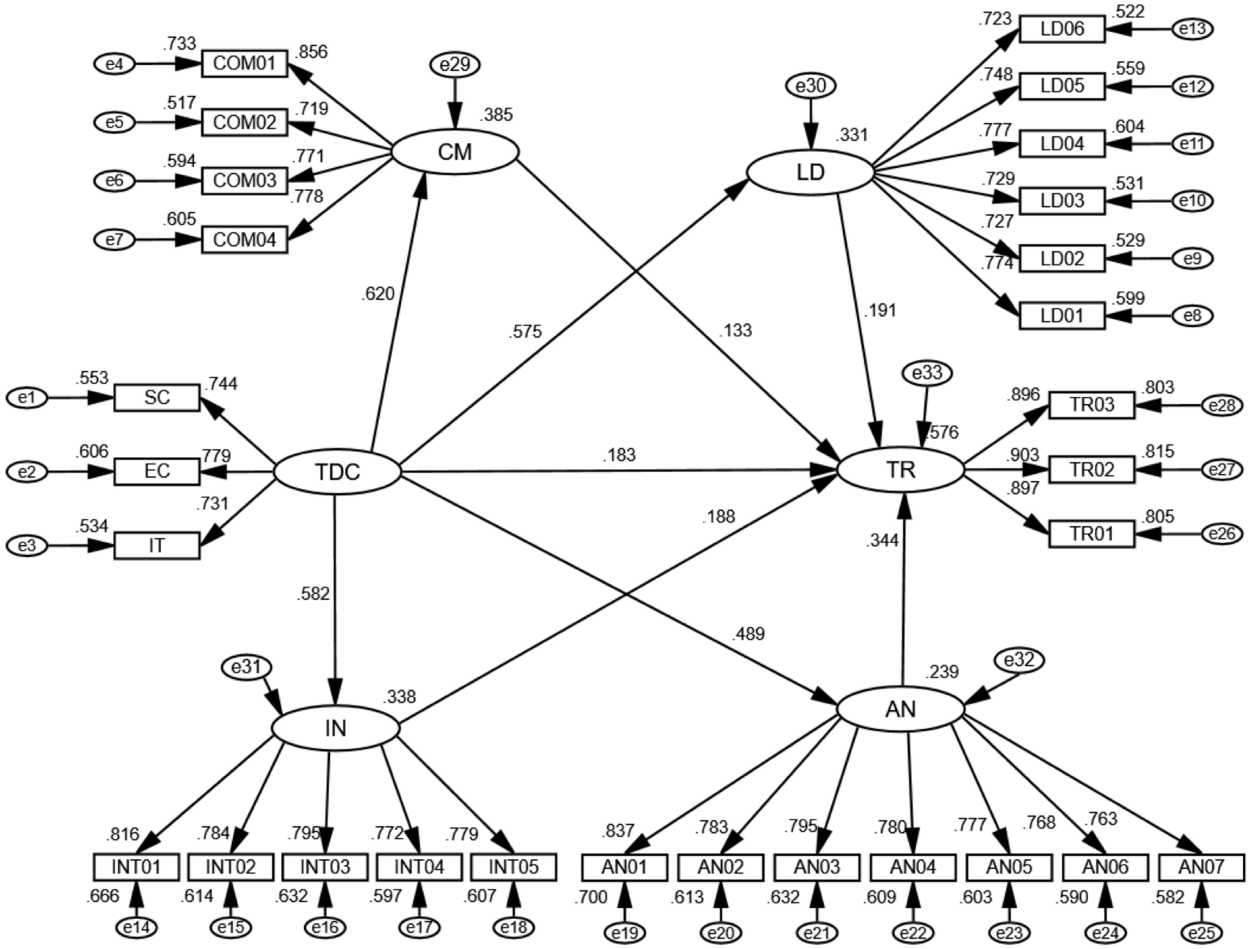
Structural model showing the standardized path coefficients among variables.

A six-factor model was constructed to analyze the overall construct validity of the different variables. The structural equation paths are shown in Figure 2.

Among the results of the path model fit indices, the RMSEA value was 0.031 and the SRMR value was 0.057, both of which are below 0.08. For the parsimonious fit index, the CMIN/DF value was 1.590, within the acceptable range of 1 to 3. With respect to the incremental fit indices, the IFI, TLI, and CFI values were all above 0.9. All fit indices were within acceptable ranges, indicating that the path model fit was acceptable.

The results of the path effects are shown in Table 4. Specifically, the independent variable team dynamic capability (TDC) had affected on communicative (CM) (*β* = 0.620, *p* < 0.001), therefore, Hypothesis H1 is supported. Team dynamic capability also affects leadership (LD) (*β* = 0.575, *p* < 0.001), therefore, hypothesis H2 is supported. Similarly, team dynamic capability (TDC) affects interpretation (IN) (*β* = 0.582, *p* < 0.001), hypothesis H3 is supported, and team dynamic capability (TDC) affects analytical (AN) (*β* = 0.489, *p* < 0.001), hypothesis H4 is supported.

**Table 4:** Structural model showing the standardized path coefficients among variables.

Path	Estimate (*β*)	S.E.	C.R.	*p*	Label
TDC → CM	0.620***	0.092	12.089	<0.001	H1
TDC → LD	0.575***	0.076	11.167	<0.001	H2
TDC → IN	0.582***	0.09	11.724	<0.001	H3
TDC → AN	0.489***	0.087	10.181	<0.001	H4
CM → TR	0.133**	0.058	2.964	0.003	H5
LD → TR	0.191***	0.066	4.527	<0.001	H6
IN→TR	0.188***	0.054	4.482	<0.001	H7
AN→TR	0.344***	0.05	8.873	<0.001	H8
TDC → TR	0.183**	0.162	2.623	0.009	–

***p < 0.001; **p < 0.01; *p < 0.05.

Furthermore, communicative (CM) affected on team resilience (TR) (*β* = 0.133, *p* = 0.003), and hypothesis H5 was supported. Leadership (LD) affected team resilience (TR) (*β* = 0.191, *p* < 0.001), and hypothesis H6 is supported. Interpretation (IN) affected team resilience (TR) (*β* = 0.188, *p* < 0.001), hypothesis H7 was supported, and analytical (AN) affected team resilience (TR) (*β* = 0.344, *p* < 0.001); thus, hypothesis H8 was supported.

According to the path results, the independent variable of team dynamic capability (TDC) has a significant positive effect on the dependent variable of team resilience (TR) (*β* = 0.183, *p* = 0.009). To further investigate whether the four variables communicative (CM), leadership (LD), interpretation (IN), and analytical (AN) play indirect roles in the relationship between team dynamic capability and team resilience, each of these 4 variables was tested as a mediator to examine the mediating effects as shown in Table 5.

**Table 5:** Mediating effects of team dynamic between team dynamic capabilities and team resilience.

Path	Estimate	Sobel		Bootstrap 95%				Sig(*p*)
				Percentile method		Bias-corrected Percentile method		
		S.E.	Z	Lower	Upper	Lower	Upper	
TDC → TR	0.183	0.075	2.440	0.042	0.337	0.042	0.337	0.012
TDC → CM → TR	0.082	0.035	2.343	0.012	0.149	0.013	0.151	0.020
TDC → LD → TR	0.110	0.029	3.793	0.056	0.171	0.057	0.173	<0.001
TDC → IN→TR	0.109	0.030	3.633	0.047	0.168	0.051	0.172	0.001
TDC → AN→TR	0.168	0.025	6.720	0.121	0.221	0.123	0.222	<0.001

For the results of the mediating effect of communicative, the indirect effect value was 0.082, the significance level Sig (*p*) was 0.020, which is less than 0.05, and the Z score from the Sobel test was 2.343, which is greater than 1.96. These results indicate that the mediating effect of communicative is significant. Thus, hypothesis H9 is supported. For the results of the mediating effect of leadership, the indirect effect value was 0.110, the significance level Sig (*p*) was less than 0.001, and the Z score from the Sobel test was 3.793, which is greater than 1.96. These results indicate that the mediating effect of leadership is significant. Thus, hypothesis H10 is supported. For the results of the mediating effect of interpretation, the indirect effect value was 0.109, the significance level Sig (*p*) was 0.001, which is less than 0.05, and the Z score from the Sobel test was 3.633, which is greater than 1.96. These results indicate that the mediating effect of interpretation is significant. Thus, hypothesis H11is supported. For the results of the mediating effect analytical, the indirect effect value was 0.168, the significance level Sig (*p*) was less than 0.001, and the Z score from the Sobel test was 6.720, which is greater than 1.96. These results indicate that the mediating effect of the analytical is significant. Thus, hypothesis H12 is supported.

## 5 Conclusions and discussion

Organizational adaptability and knowledge-driven innovation capability have become critical to the development of higher education institutions. Faced with complex reform tasks and strategic transformation goals, team dynamic capabilities—particularly in terms of cross-functional collaboration, knowledge integration, and adaptive decision-making—are of vital importance in universities. Investigating dynamic capabilities within university teams contributes to a deeper understanding of how local Chinese universities enhance team functioning to respond effectively to institutional reforms and resilience challenges under policy-driven contexts.

Previous research has primarily examined team resilience from the perspective of resource conservation, focusing on the accumulation and management of existing resources. This study shifts the focus to the dynamic capability mechanisms underlying team resilience, exploring how the knowledge creation process—considered a core element of team dynamic capability—influences resilience. Particular attention is given to the functional aspects of team dynamics. The findings reveal that communicative, leadership, interpretation, and analytical jointly form the dynamic functioning of teams, acting as partial mediators and significantly enhancing the practical value of knowledge within teams.

### 5.1 Theoretical implications

First, by focusing on the dynamic processes of teams, this study deepens the understanding of team resilience in the context of organizational change and clarifies theoretical boundaries by proposing a shift from a “resource-preservation” perspective to a “functional-evolution” perspective. As noted by Galy et al. (2023), Brykman and King (2021), since 2011, much of the literature has conceptualized resilience based on the Conservation of Resources (COR) theory (Hartmann et al., 2020; Hartwig et al., 2020; Hobfoll, 2011; Hobfoll et al., 2018; Stoverink et al., 2020; Westman, 2001), portraying resilience as a static attribute and a key mechanism for linking and protecting various resources through intersecting processes. This view primarily supports the restorative function of resilience. However, in relatively stable yet complex transformation contexts—often described as brittle, anxious, non-linear, and incomprehensible (BANI) systems (Cascio, 2020)—resilience increasingly entails the accumulation of resources, such as knowledge (Cascio, 2020), and is better understood as a dynamic function that supports sustainability and adaptive development in response to multifaceted challenges. Against this backdrop, the present study adopts a dynamic capability perspective, emphasizing how teams engage in sensing, seizing, and reconfiguring to proactively adapt to change. This reconceptualizes resilience as an evolutionary outcome rather than merely a restorative attribute, offering a novel theoretical lens for understanding team resilience.

Second, this study explores how resilience emerges through the interplay of multiple causal mechanisms by investigating the mediating effects among key behavioral constructs. As previously noted, although one of the foundations of resilience lies in the accumulation of knowledge resources, Nonaka and Takeuchi (2021) emphasize that while knowledge creation enables teams to acquire new insights, it alone does not confer the wisdom necessary for applying knowledge effectively. The true value of knowledge is realized through “knowledge practice,” that is, through the application, utilization, dissemination, and transformation of knowledge into action. Building on this premise, this study identifies communicative, leadership, interpretation, and analytical capacities as critical behavioral capabilities that collectively function as mediating mechanisms in the transformation of dynamic capabilities into resilience. This mechanistic perspective not only deepens the understanding of how dynamic capabilities contribute to resilience but also echoes Duchek (2020) view that resilience depends on the interaction between cognitive and behavioral capacities and organizational action. Furthermore, this behavioral pathway aligns with Zollo and Winter (2002) theory of organizational capability evolution, which emphasizes the gradual development of capabilities through mechanisms such as experiential learning, knowledge articulation, and codification. By uncovering the mediating behavioral pathways, this study provides a more actionable theoretical foundation for understanding the mechanisms that enhance team resilience.

### 5.2 Practical implications

First, it is essential to encourage knowledge creation processes within teams. As knowledge-based institutions, universities rely heavily on productivity and the effective generation of knowledge (Nkambule et al., 2024). In the context of innovation and change, high-quality development in universities involves the activation, reorganization, and systematization of knowledge. The dynamic capability of knowledge creation enables teams to aggregate differentiated knowledge, allowing members to efficiently and innovatively accumulate knowledge within a short time frame, even as their individual domains of expertise become more specialized. Team members can respond to complex environments by developing diverse skills, behaviors, and attitudes that collectively enhance the team’s capacity to address environmental challenges and produce valuable outcomes. Moreover, for universities and their working teams, managers should place team collaboration at the core of knowledge creation, emphasizing the participatory design of team members. Collective creation should be leveraged as a means of knowledge mobilization—enabling the sharing, activation, and utilization of available knowledge (Langley et al., 2018). This approach reflects the team’s capacity to sense its environment, capture advantageous information, and reconstruct it meaningfully. For example, institutions may establish interdisciplinary workshops or innovation labs, and develop dedicated knowledge integration platforms for teaching and research teams, or collaborative platforms for administrators. These platforms can systematically document team members’ expertise, interests, progress, and resource needs, thereby enhancing the efficiency of knowledge sharing and mobilization.

Second, it is crucial to prioritize the development of team dynamic capabilities within universities. In the practice of knowledge management in academic teams, it is important to recognize that the process of developing dynamic capabilities is influenced by collective functional mechanisms. University team management should acknowledge that dynamic capabilities are shaped and sustained by structured collective processes. Research teams engaged in organized scientific activities are expected to demonstrate collective intelligence in their outputs. Accordingly, team members must possess lifelong learning abilities, effective interpersonal communication skills (Aziz et al., 2024), critical thinking, and strong knowledge management competencies (Ding et al., 2019).

Third, as team resilience is considered a form of shared consensus outcome (Chapman et al., 2020; Raetze et al., 2021), strengthening team resilience can enhance problem-solving and decision-making quality within university management teams, as well as foster innovation in management processes. Given the current strategic importance of digital transformation (DT), resilience-building plays a pivotal role in guiding and enabling such transitions (Benavides et al., 2020; Rof et al., 2020). Emphasizing contingency planning and reflective mechanisms within teams requires members to document daily management processes, encourages continuous learning, and fosters anticipation of change, thereby enabling team members to become more adaptable, stable, and self-reliant (Shaya et al., 2023).

### 5.3 Limitations and potential research directions

Although this paper provides constructive insights into team resilience, several limitations should be acknowledged. The research model is grounded in the context of local public universities, which restricts the generalizability of the findings due to the singular organizational type and cultural background. It does not account for the hierarchical structure of Chinese higher education institutions (e.g., national vs. local universities), nor does it test the applicability of the model across industries (e.g., business, nonprofit sectors) or cultural settings (e.g., high vs. low power distance societies). These contextual differences are increasingly recognized as both limitations and opportunities for future cross-cultural research. Future studies should consider expanding the sample scope to include a broader range of institutional types and geographic regions. This would allow for an exploration of how dynamic capability configurations function differently across various sectors and cultural environments, thereby contributing to the development of a more universally applicable theoretical framework of team resilience.

This paper employed a serial mediation model to represent the dynamic functions of teams, which remains exploratory in nature. The proposed sequence is not intended to be definitive or exhaustive; rather, it reflects a theoretically grounded configuration informed by the literature on knowledge creation, sensemaking, and team learning (e.g., Nonaka et al., 2016; Weick, 1995; Edmondson, 1999). Future research could test alternative models, such as adopting the Knowledge-to-Action Framework (KTA) (Graham et al., 2006) as a comprehensive theoretical foundation. Additionally, incorporating contextual moderators may further refine the mechanisms through which team dynamic capabilities influence resilience, thereby contributing to the development of a more robust theoretical model.

In line with Farnese et al. (2019), the cross-sectional design precludes the ability to make statements of causal relationships on organizational performance indicators, and future studies utilizing time-lagged or multisource data are recommended. The findings of this study reflect individual-level perceptions rather than objectively constructed team-level phenomena. Future research should adopt multilevel designs with appropriate aggregation indices to more accurately capture team-level constructs. Additionally, the integration of team dynamic capability with other variables in a combined model was intended to assess discriminant validity. Although hierarchical modeling might be more conceptually appropriate, technical constraints in addressing discriminant validity at the single-dimension level limited this approach. Future research may explore the use of hierarchical modeling followed by integration into a comprehensive structural model.

## Data Availability

The raw data supporting the conclusions of this article will be made available by the authors, without undue reservation.
